# Splenic infarction during *Plasmodium ovale *acute malaria: first case reported

**DOI:** 10.1186/1475-2875-9-288

**Published:** 2010-10-18

**Authors:** Gaël Cinquetti, Frédéric Banal, Candyce Rondel, David Plancade, Charlotte de Saint Roman, Dina Adriamanantena, Céline Ragot, Serge Védy, Bruno Graffin

**Affiliations:** 1 Service de Médecine Interne et Maladie Infectieuses et Tropicales, Hôpital d'Instruction des Armées Legouest, 27 avenue de Plantières, BP 90001, 57077 METZ Cedex 3, France; 2 Service de Réanimation, Hôpital d'Instruction des Armées Legouest, 27 avenue de Plantières, BP 90001, 57077 METZ Cedex 3, France; 3 Service de Chirurgie Viscérale, Hôpital d'Instruction des Armées Legouest, 27 avenue de Plantières, BP 90001, 57077 METZ Cedex 3, France; 4 Service de Biologie, Hôpital d'Instruction des Armées Legouest, 27 avenue de Plantières, BP 90001, 57077 METZ Cedex 3, France

## Abstract

The splenic complications of acute malaria include two different prognostic and treatment entities: splenic infarction and splenic rupture. This is the first case of splenic infarction during an acute malaria due to *Plasmodium ovale *in a 34-year-old man. As in the majority other described cases of splenic infarction, the course was spontaneously favourable, suggesting that this complication was relatively benign compared to splenic rupture, which is life-threatening and usually necessitating surgery.

## Background

Splenic infarction is a rare complication usually described during acute malaria with *Plasmodium falciparum *or *Plasmodium vivax*. Splenic rupture is another, less well-known life-threatening malaria complication. A case of splenic infarction during acute malaria due to *Plasmodium ovale *is reported. This is the first case described in the literature with this species. This case supplements the splenic complication panel of acute malaria, with two different prognostic and treatment entities: splenic infarction and splenic rupture.

## Case report

A 34-year-old man, a French professional soldier, with no remarkable medical history, had two previous exposures to endemic malaria zones (four months in Senegal in 2002, and four months in Ivory Coast in 2004). He stated having taken his anti-malarial chemoprophylaxis (doxycycline, 200 mg/day) more or less regularly during his stays, having omitted to take the medication on several occasions. He never had acute malaria previously.

In 2008, he developed a 39,5°C fever with headache, asthenia, myalgias, with nausea and vomiting. After a few days of self-treatment with paracetamol, he presented to the Emergency Department at the Military Hospital Legouest, Metz, France. Physical examination showed a 14 cm painless splenomegaly, and a temperature of 39°C. All other physical findings were normal. Laboratory tests showed anaemia (Hb 11.8 g/dl), thrombocytopaenia (platelets 117×109/mm3), leukopaenia (3250/mm3), lymphocytopaenia (839/mm3) and a moderate increase in liver enzymes (aspartate aminotransferase/alanine aminotransferase were both about twice the upper limit of normal values). The diagnosis of acute malaria was made by blood smear examination, with 0.001% *P. ovale *parasitaemia. The species diagnosis was confirmed by molecular biology (polymerase chain reaction), without bi-parasitism (no *P. falciparum *or *P. vivax *associated).

The patient was admitted and treatment was started with intravenous quinine because of vomiting (8 mg/kg three times a day), and was well tolerated. Fever subsided gradually over three days, anaemia resolved without transfusion and signs of hepatic cytolysis disappeared within a few days.

**Figure 1:**
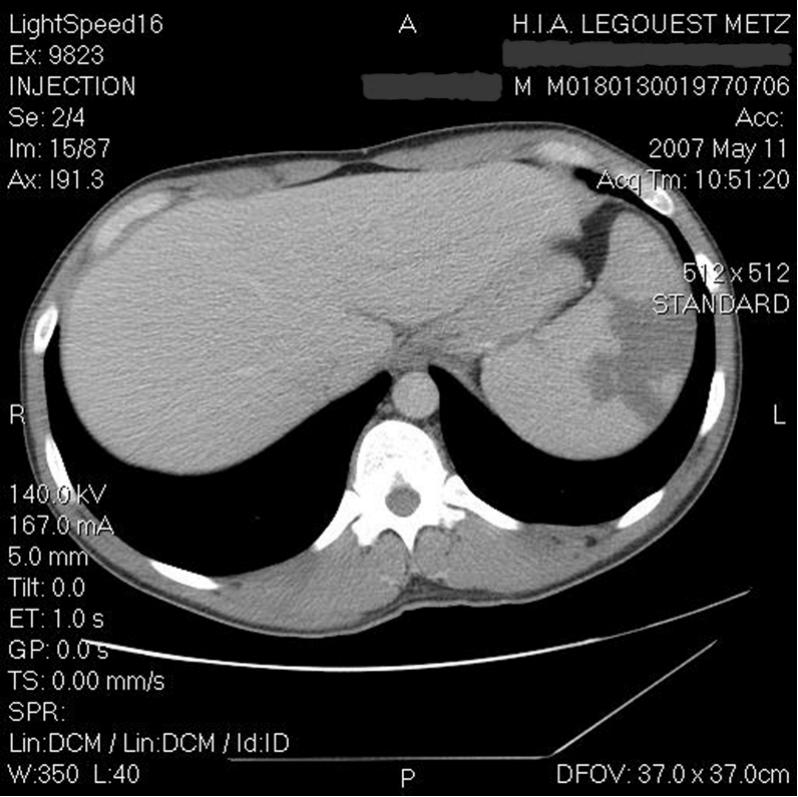
**Abdominal CT-scan: splenomegaly and splenic infarction**.

Six days after his admission, the patient complained of a sudden onset of left hypochondrial pain. Physical examination showed persistent, painful and tender splenomegaly. Abdominal CT-scan (Figure 1) showed several splenic infarcted zones, the largest measuring 53 × 37 mm, in the upper pole in the anterior edge, and in the dorsal face as well as in the lower pole.

His haemodynamic status was stable and simple analgesics were effective, obviating the need for emergency surgery. Management consisted only of medical monitoring and analgesia (subcutaneous morphine).

The splenic abnormalities regressed spontaneously over several months, during which the patient continued to tire easily. Haemoglobin was 10.6 g/dl one month after initial onset. Exploration of other causes of splenic infarction, including constitutional underlying coagulation disorder (C and S-protein, Leyden mutation, homocystein, antithrombin III and antibody antiphospholipid measurement), sickle cell disease (normal haemoglobin electrophoresis) and concomitant Salmonella infection, were negative.

## Conclusions

Malaria is one of the main infectious threats for the French army. Engaged in short-term missions or outside operations, 35,000 to 40,000 French soldiers stay every year in zones of endemic malaria disease. For 10 years, the rate of malaria incidence in the French army has been between 1,9 and 5,8 percent of men annually, and unfortunately, four deaths have been reported.

In 2008, 413 acute malaria cases were declared in the French army. 92,6% of which were uncomplicated clinical forms. 56.4% of the cases were due to *P. vivax*, 36% to *P. falciparum*, 1.2% to *Plasmodium malariae *and 6.3% to *P.ovale*, the frequency of which is on the increase.

Splenic infarction is a rarely described, and probably under-diagnosed, complication of malaria. Bonnard *et al *[1] found only eight documented cases of splenic infarction associated with malaria in the literature. Two other case reports are described, including a case discovered fortuitously during an autopsy, adding up to a total of ten descriptions. Frequently, they were young patients (3 to 30 years old), all infected with *P. falciparum *(except one case of co-infected with *P. vivax *and one case unknown), without anti-malarial prophylaxis (except one, and two unknown). They all survived with medical treatment (except one surgical treatment). No death due to splenic complications is mentioned.

Rupture of the spleen is more common than splenic infarction during malaria and is a diagnostic emergency, usually necessitating surgery. Pathological rupture of the spleen has rarely been reported following *P. ovale *or *P. malariae *infection. In a recent litterature analysis, Imbert *et al *[2] found only two cases involving *P. ovale*. This is a very serious, life-threatening complication, even if the two cases described show that the issue was not always fatal.

Infarction-induced haemorrhage does not consistently fit available pathological data. Haemorrhages have been observed in splenic tissue devoid of signs of infarction, and infarction was noted in only two of 55 spleen rupture cases reported over the last 50 years.

In the 10 cases of splenic infarction reported over the last 30 years, only two cases were complicated by splenic rupture (in a child with a *P. vivax *and in a *P. falciparum *mixed infection and in an adult suffering from *P. vivax *malaria) [3,4]. For this reason, splenic infarction must be considered as a different complication from splenic rupture in acute malaria, with a very different prognostic and treatment. This complication must be evoked whatever the plasmodial species. As in the majority other described cases, the course was spontaneously favourable, suggesting a relative benignity of this complication.

## Consent

Written informed consent was obtained from the patient for publication of this case report and any accompanying images. A copy of the written consent is available for review by the Editor-in-Chief of this journal.

## Competing interests

The authors declare that they have no competing interests.

## Authors' contributions

GC, FB and CR wrote the manuscript. DP, CSR, DA, and BG carried out the physical examination. CR and SV carried out the biological analysis. All authors read and approved the final manuscript.
